# Cardiac–Brain Dynamics Depend on Context Familiarity and Their Interaction Predicts Experience of Emotional Arousal

**DOI:** 10.3390/brainsci12060702

**Published:** 2022-05-29

**Authors:** Sudhakar Mishra, Narayanan Srinivasan, Uma Shanker Tiwary

**Affiliations:** 1 Indian Institute of Information Technology Allahabad, Prayagraj 211012, India; ust@iiita.ac.in; 2 Indian Institute of Technology Kanpur, Kanpur 208016, India

**Keywords:** cardiac–brain interaction, context familiarity, naturalistic paradigm, mixed effect modelling, emotional film, emotional arousal, interoception

## Abstract

Our brain continuously interacts with the body as we engage with the world. Although we are mostly unaware of internal bodily processes, such as our heartbeats, they may be influenced by and in turn influence our perception and emotional feelings. Although there is a recent focus on understanding cardiac interoceptive activity and interaction with brain activity during emotion processing, the investigation of cardiac–brain interactions with more ecologically valid naturalistic emotional stimuli is still very limited. We also do not understand how an essential aspect of emotions, such as context familiarity, influences affective feelings and is linked to statistical interaction between cardiac and brain activity. Hence, to answer these questions, we designed an exploratory study by recording ECG and EEG signals for the emotional events while participants were watching emotional movie clips. Participants also rated their familiarity with the stimulus on the familiarity scale. Linear mixed effect modelling was performed in which the ECG power and familiarity were considered as predictors of EEG power. We focused on three brain regions, including prefrontal (PF), frontocentral (FC) and parietooccipital (PO). The analyses showed that the interaction between the power of cardiac activity in the mid-frequency range and the power in specific EEG bands is dependent on familiarity, such that the interaction is stronger with high familiarity. In addition, the results indicate that arousal is predicted by cardiac–brain interaction, which also depends on familiarity. The results support emotional theories that emphasize context dependency and interoception. Multimodal studies with more realistic stimuli would further enable us to understand and predict different aspects of emotional experience.

## 1. Introduction

Most lab-based emotion research is conducted with static stimuli and paradigms that have less ecological validity. Recently, there has been a shift towards the use of more naturalistic stimuli to probe the neural dynamics of emotions [1]. The stimuli used in such studies include virtual reality-based immersive experiences [2], emotional movie clips [3], and emotionally salient films [4]. These stimuli offer a better probe to track task-related neural responses, autonomic responses, and subjective feedback in a realistic and dynamic environment. Our current study probes the relationship among familiarity, arousal, neural, and cardiac responses during the emotional experiences with film stimuli.

ECG signal is mainly comprised of P-QRS-T complexes and can be considered a composite signal with many frequency components that arise from various physiological processes. Thus, subtle changes in these physiological processes reflect changes in the P-QRS-T complexes during each cardiac cycle and also reflect power at different frequency components of the ECG signal [5]. The spectral content of the T wave, P wave, and QRS complex is generally within a range from 1 to 10 Hz, 5 to 25 Hz, and 8 to 50 Hz [6], respectively. Changes in the high-frequency range (above 40 or 50 Hz) are used to probe clinical conditions, such as myocardial ischemia [6,7]. Here, we focus on frequencies below 40 Hz, given that the spectral content of the T wave and P wave are reported to be confined to the lower frequency range (1–25 Hz) [6,8]. The frequency range 25 to 40 Hz is generally linked to the QRS complex in ECG. A recent study linked the power spectrum of ECG with cardiac activity such that the magnitude of frequencies in the power spectrum of ECG depends on HRV. The lower the HRV, the larger the magnitudes of peaks, resulting in more power along with the increasing number of distinct frequency peaks [5]. Hence, it is likely that subtle changes in cardiac dynamics during the emotional experience could be reflected in the power spectrum of the cardiac signal in the midF range.

The brain controls the rate, rhythm, and power of heartbeats via both sympathetic (’fight and flight’) and parasympathetic (’rest and digest’) axes of the autonomic nervous system [9]. In addition, cardiac functions can be profoundly altered by the reflex activation of cardiac autonomic nerves in response to central autonomic commands associated with stress, physical activity, arousal, and attention [10]. On the other hand, the beat-by-beat operation of the heart is also tuned to engage the emotional and cognitive resources [11]. The afferent cardiac signals facilitate information gathering [12] and modulate sensory attention to perceive the low spatial frequency features of fearful faces [13]. The cardiac activity, mediated through the amygdala, facilitates fine-grained discrimination at the sensory cortices [14] and promotes active sampling to resolve uncertainty [15]. In a recent resting-state study, frontal cortex activity has been linked to the modulation of cardiac activity with reduced activity in the prefrontal delta and theta power correlated with higher cardiac vagal control [16]. Moreover, it has been argued that the physiological interrelation between the heart and brain influences emotional experience. The dynamic, continuous, and bidirectional communication of both organs influences one’s perception, and emotion [17,18]. The physiological coherence model based on bi-directional communication pathways postulates the afferent and efferent pathways between heart and brain and describes state-specific emotions, the role of the body in resetting behavioural patterns, and the changes in psychophysiological systems in response to self-induced positive emotions [19].

Emotion-related EEG studies have attempted to understand the role of different frequency bands and brain regions in emotion processing [2,20,21,22]. For instance, synchronized gamma band activity in the prefrontal (PF) cortex is reported for the emotional information processing while watching emotional film clips, especially activity in the left frontal cortex [20]. Unpleasant stimuli increased gamma power in the frontal regions as well as large-scale gamma phase synchronization across frontal regions [23]. High gamma activity is also recorded during increased emotional responses in a cognitive reappraisal study with affective pictures [24]. Activation of the right PF has been linked to a rise in cortisol when emotional stimuli were presented [25]. A high cortisol level is linked to emotional arousal. The activity in the PF regions is also linked with cardiac activity. Throughout the resting condition, the activation/deactivation of the PF regions has been observed to modulate the inter-beat-interval through the vagal control of the heart rate [16]. Gamma band activity in the left PF regions was observed in the processing of the emotional information of emotional film clips [20].

Recent studies have argued that interoceptive predictions about feelings contribute to emotion perception and feelings [26,27,28]. Accumulating evidence indicates that the interoceptive experience may reflect the visceral predictions about these feelings, adjusted to accommodate the current sensation [10,12,13]. A heart-evoked potential (HEP) in frontocentral (FC) electrodes is observed during heartbeat detection compared to the resting condition, possibly due to activity in the interoceptive network in the frontocentral regions [21]. Other than activity in the frontal cortex, interoceptive activity has also been reported in the parietooccipital (PO) region [22]. For instance, spectral power in the alpha band over PO regions was higher while attending to the heart compared to the visual task and was associated with the subjective performance in the interoceptive task [22]. With an ecologically valid experimental setting, the experience of arousal during a virtual reality experience of the rollercoaster ride was associated with the activity in the parietooccipital (PO) electrodes in the alpha band [2]. Hence, in this exploratory study, we focused on the PF, FC, and PO regions to investigate the relationship between cardiac and brain activity during the processing of naturalistic emotional stimuli potentially influenced by the context familiarity.

Appraisal theorists describing emotion mechanisms have argued that the degree of context familiarity is an essential determinant of the nature of affect elicited in a particular situation [29]. The appraisals can be fairly quick or automatic associations matching with the patterns in the environment. Depending on these subjective associations, people could have different affective responses to the same situation [29]. Other theories describing the familiarity–affect relationship, including behavioural inhibition system theory (BIS) [30], entropy model of uncertainty [31], and fear of unknown theory [32], have one thing in common in that they all view lower familiarity as a deficit of knowledge that is inherently aversive. The lower familiarity can be mediated by several factors, including information seeking and the subjective perception of lack of information [33]. Although the importance of familiarity is well recognized in theories of emotion, the effect of familiarity on central and autonomic activity during an emotional experience is not comprehensively explored. To the best of our knowledge, no study to date probed the influence of familiarity on cardiac–brain interaction while emotion is being felt.

Constructivist theories of emotion also argue that felt emotions are based on context [29]. Context helps in situating the changes in interoceptive signals in the conceptual framework of emotions. The internal bodily sensations are used to categorize emotional experiences [34]. These categorical associations are influenced by cultural context, translating the interoceptive experience into a social concept called emotions [27,35]. The learnt contextual descriptors reflect how these interoceptive signals are interpreted emotionally, and these descriptors also introduce variety in interpreting the same interoceptive signal [35]. Hence, coupling the physiological changes with the emotional experience is central to the theories advocating an interoceptive basis for emotional feelings [36].

Several studies have explored the coupling of physiological changes with emotional experience. For example, participants with high interoceptive accuracy reported a higher correlation between their heart rate reactions and subjective arousal for negative images from IAPS [37]. Recently, a study recorded skin conductance responses of participants looking at happy and fearful emotional facial expressions [38]. The higher sensibility to interoceptive changes was related to the low reaction time in recognizing emotions from facial expressions (particularly emotional faces with high arousal). In line with these results, an fMRI study [39] also reported emotional priming by interoceptive signals in recognizing emotional facial expressions. In an ERP study, the participants who were better heartbeat perceivers experienced higher arousal for emotional pictures (highly arousing IAPS pictures for pleasant and unpleasant emotions) and showed higher amplitude for emotional pictures at the antero-inferior, medial, and posterior electrode sites [40].

The effect of expectation or familiarity on interoceptive (cardiac) activity and emotions has been primarily investigated using a repeated suppression paradigm with pictures as stimuli [41,42,43]. For instance, the expected and unexpected emotional facial expressions (angry expressions) influenced the heartbeat evoked potential (HEP). The study reported a positively deflected HEP waveform in the FC region for the unexpected negative facial expressions [42]. Another EEG study probed the effect of priming cues to form expectations regarding the upcoming (neutral or angry) facial expressions [43]. The HEP amplitude following R-wave was correlated with the neural responses in FC electrodes for the cued task. On the other hand, the visual evoked potential (VEP) was correlated with the central-parietal and parietal electrodes. It should be noted that the above-mentioned studies are performed with emotional pictures and not dynamic stimuli.

Given the putative link between interoception and emotional experience (specifically emotional arousal) and the interplay between cardiac and brain activity with the effect of familiarity using emotional pictures, our purpose of conducting this exploratory study was threefold. First, the nature of cardiac–brain interaction during emotional feelings with naturalistic stimuli is not well understood. Hence, we used film clips to elicit and mark the time of emotional experience to understand cardiac–brain dynamics just before the experience. More importantly, if both interoception and cue to the upcoming stimuli play an essential role in our emotional experience (as reported in the past studies with static stimuli), then there is the possibility that the familiarity of the emotional context would interact with cardiac activity to probably influence our brain activity during emotional experience. Third, emotional arousal could be linked to the familiarity of the emotional stimuli as well as cardiac and neural activity.

## 2. Materials and Methods

### 2.1. Participants

Forty-three participants were recruited from the Indian Institute of Information Technology, Allahabad. Three participants were excluded from the analysis due to excessive movement. The final sample thus consisted of forty individuals (three females) with mean age (23.3±1.4). Volunteers obtained course credits for their participation. All participants had normal or corrected-to-normal vision and no known neurological or mental disease history. Inclusion criteria for all participants were based on Axis I assessment, defining the criteria for mental health [44]. No participant was excluded based on their DSM ratings. All participants provided written informed consent for the study according to the Helsinki declaration, and the study was approved by the Ethics Committee of the Institute (IERB ID:2017-100).

### 2.2. Stimuli

Stimuli for emotion elicitation were selected from the affective stimuli dataset that we had developed (for stimuli [45]; for related article [46]). The following criteria were applied for the selection of stimuli: (1) high probability of emotion elicitation and good concordance score based on our earlier ratings study [45], (2) at least one stimulus from each emotion category, and (3) more preference to videos from India. The set of selected stimuli are listed in Table A1. The time duration of each stimulus is 60 s. Each participant was shown nine emotional stimuli selected randomly from the set of 15 stimuli.

Non-emotional videos were separately validated with 15 participants. Eight non-emotional videos were shown to participants, and two of them were selected. However, in this work, we did not analyse the non-emotional videos and data related to only emotional videos were analysed.

### 2.3. Self-Assessment Scales and Presentation of Emotional Categories

We used six self-assessment rating scales to obtain the ratings of subjective experience. The valence scale varies from unpleasant (1) to pleasant (9). Self-Assessment Manikin (SAM) pictures depicting unpleasant to pleasant faces were shown above the rating scale to help participants rate valence. The arousal scale ranged from inactive (1) to active (9). Again, SAM pictures depicting inactive or low arousal to active or high arousal were shown above the rating scale. The dominance scale ranged from submissive (1) to dominant (9). The SAM picture depicted the range of dominance with the size of cartoons. How much participants liked a stimulus was rated in the liking scale ranging from least (1) to fair (3) to much (5). The familiarity of the content was rated on the familiarity scale ranging from less-familiarity (1) to high-familiarity (5). Participants were instructed to rate the familiarity scale as follows. ’While rating the familiarity scale, recall how well and frequently you were able to predict what could be the next event or episode or scene in the stimulus. According to this self-judgement, you have to rate the familiarity scale from 1 (less familiarity) to 5 (high familiarity)’. The last scale was the relevance scale, which was rated based on the assessment of any personal past life event that resembles the situation shown in the stimulus video. This continuous scale ranged from not related (1) to completely related (5). Emotional categories were presented in the form of a drop-down menu.

### 2.4. Experiment

The experiment was performed in a dark room. The chair with armrest was immovable, and the screen was kept at a 1-meter distance from the participant. A dedicated computer system presented the stimuli on a 15.6-inch screen display at a reduced resolution of 800 × 600. Audio information was given using Sennheiser CX 180 Street II in-Ear headphones. Participants were allowed to adjust the audio volume to their comfort level during training. They responded using the mouse and keyboard.

A Mac-based high density (128-electrode HCGSN; Net Amps 400) EEG system from EGI was used to record the EEG signals. The EEG cap was placed on the participant’s head, and the impedance was kept under 30Kohm. ECG signal was acquired using the physio 16 PNS box from EGI. The right and left ECG electrodes were placed directly below the clavicle near the right and left shoulder. Netstation software was used to acquire the raw signal at 250 Hz sampling rate.

**Figure 1:**
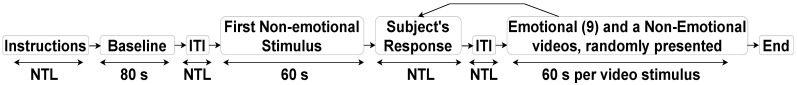
Experiment: EEG experimental paradigm. NTL: no time limit; ITI: inter-time interval.

The experimental paradigm is shown in Figure 1. Participants were provided instructions and trained using three video clips for the emotional task. The experimenter discussed and provided any needed clarifications to the participants. Information about scales was presented followed by the baseline recording with eyes open (participants were looking at the fixation cross) for 80 s and presentation of emotional and non-emotional video trials. A trial consisted of a video clip, self-assessment scales, and selection of emotion categories.

The experiment started with a trial consisting of the presentation of a non-emotional video. Subsequently, ten trials with one video in each trial were presented. There were nine emotional and one non-emotional videos presented in random order. The duration of each video was 60 s. The trial with the second non-emotional video occurred anywhere after the fifth trial and before the eighth trial. In each trial, the presentation of a video was followed by the presentation of self-assessment scales including valence, arousal, dominance, liking, familiarity, and relevance. Participants rated their assessment of subjective feelings on these scales. Critical aspect of the design is that the design allowed participants to click their mouse and indicate that they were feeling a particular emotion while watching the video. This enabled us to record the time at which they felt an emotion. After rating the self-assessment scales for each stimulus, participants labelled each mouse click with an emotion category suitable to their emotional feelings. Participants were shown three frames, extracted around the mouse clicks to help recall their felt emotion and labelled them. During the inter-trial interval, a fixation cross with simple mathematical addition (optional and mental) was presented to reduce the effect of emotional feelings from the previous stimulus. Once the participants felt comfortable to perform the next trial, they clicked an arrow on the screen and started the next trial.

### 2.5. Pre-Processing

#### 2.5.1. EEG

**Figure 2:**
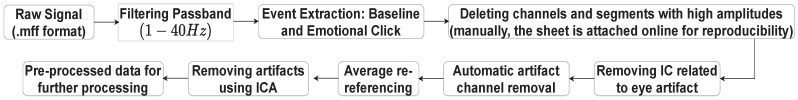
EEG Preprocessing pipeline.

The pre-processing steps are shown in Figure 2. The raw signal was imported, which was already referenced to Cz. We performed average rereferencing during the pre-processing stage. The raw EEG signal was filtered with a fifth-order Butterworth bandpass filter with the low cutoff 1 Hz and high cutoff 40 Hz. From the filtered signal, the event segments corresponding to baseline state and the clicks were extracted with time-duration 10 s to 70 s and −6 s to 1 s, respectively. The extracted duration for the click event was from 6 s before the click event to 1 s after the click.

Next, the concatenated signals were manually checked. The electrodes and samples with very high amplitude, probably caused by the detachment of electrodes, were removed manually (The manual rejection sheet is online [47]). Then, we applied Independent Component Analysis (ICA) to remove eyeblink activity. We removed the eye blink artefacts so that the electrodes would not be discarded in the automatic channel rejection step due to high amplitudes of eye artefacts. After the automatic channel rejection step, a second ICA was applied to remove any remaining artefacts in the signal (including muscle activity, heart activity, line noise, and channel noise). The ICLabel tool is used to label the independent components into the different labels mentioned above [48]. We kept independent components with the probability of brain activity greater than 0.3 (adding the probability for all the labels equal to 1). After completing the pre-processing steps, the individual events corresponding to baseline state and emotion clicks were stored in .mat files for further data analysis.

#### 2.5.2. ECG

Data from ECG electrodes were extracted and bandpass filtered with a low cutoff of 1 Hz and a high cutoff of 40 Hz (all other filter parameters are the same as the EEG pre-processing). Only segments corresponding to the time duration click events (duration as mentioned in the EEG pre-processing) were considered from the continuous signal. Three ECG recordings were deleted based on the visual inspection.

### 2.6. Power Calculation

Both EEG power and ECG were calculated using Fast Fourier Transform (FFT). The mean square of the amplitude of frequencies in five frequency bands was calculated as power. The range of the five frequency bands is as follows: theta (4–8 Hz), alpha (8–13 Hz), lower beta (13–20 Hz), upper beta (20–30 Hz), and gamma (30–40 Hz). The mean square of the amplitude of mid-frequency band (midF: 25–40 Hz) in ECG was calculated as midF ECG power.

### 2.7. Scalp Sites for Investigation

**Figure 3:**
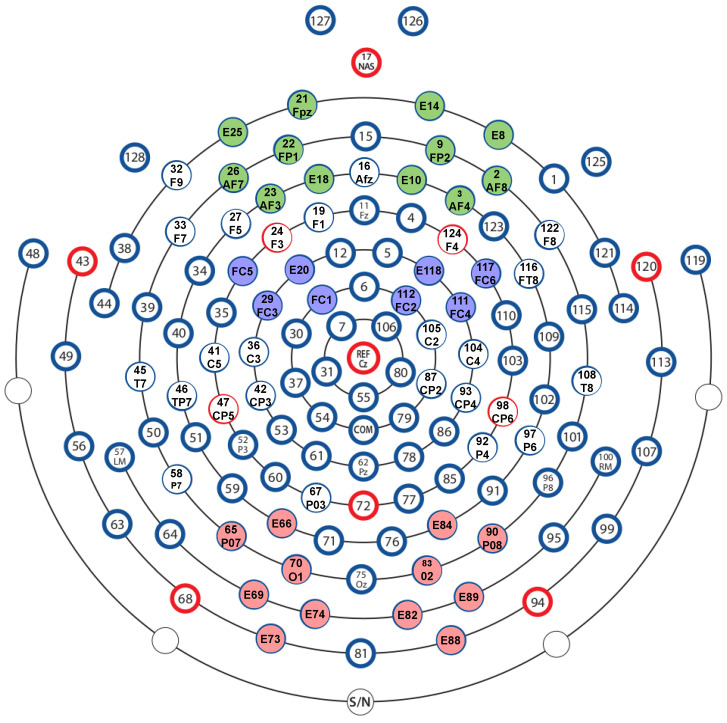
The EEG cap layout shows the electrodes considered for the analysis with colour coding: Green—Prefrontal Regions; Purple—Frontocentral Regions; Red—Parietooccipital Regions.

We investigated three scalp regions, including PF, FC, and PO. The EGI electrodes included in the left PF region were Fp1, AF3, AF7, E18, E21, E25, and in the right PF region were Fp2, AF4, AF8, E8, E10, and E14. Electrodes FC1, FC3, FC5, and E20 formed the left FC region and electrodes FC2, FC4, FC6, and E118 formed the right FC region. In the left PO region, six electrode sites were included—O1, PO7, E66, E69, E73, and E74. Electrodes in the right PO region included PO8, O2, E82, E84, E88, and E89. Figure 3 shows the layout of the EEG cap with colour annotation depicting the electrodes considered for analysis.

### 2.8. Statistical Analysis

As mentioned in Section 2.6, ECG power was calculated for the mid-frequency band (midF: 25–40 Hz). EEG signal was divided into theta, alpha, lower beta, upper beta, and gamma bands (as mentioned in Section 2.6). A set of electrodes from either the left or right hemispheres was clubbed together for three different regions as mentioned in Section 2.7. For each stimulus, the average EEG and ECG power of emotional clicks was calculated. In total, there were 235 trials for which at least one emotional click was performed. Hence, there was a total of 235 observations for 40 subjects.

We performed regression analysis using linear mixed effect modelling with EEG power as the response variable. We analysed whether the interaction between ECG power and familiarity can predict the EEG power. We tried two random effect designs; first, random intercept only within subject and second, the crossed random effect design with subject and stimulus. With our data, the crossed random effect design model had an issue of overfitting, which was checked using random effect principle component analysis (rePCA). rePCA checks the dimensionality of the random effect distribution [49] by calculating the variance–covariance matrix. We found that the co-variance of stimulus factor was zero, which indicates that the random effect structure should be one dimensional (i.e., subject factor). Hence, we did not consider stimulus as a factor because it did not seem to explain within-subject variance in our data.

The predictors’ coefficient was estimated using a restricted maximum likelihood method (REML). The significance calculation was performed using the Kenward-Roger (KR) method. KR method was recommended to evaluate the significance of mixed-effects models due to its advantage in type 1 error rates [50]. Participants were modelled as ’by-subject’ random effect for the linear mixed model to account for variance due to correlated measurements within the subject. The model selection procedure was performed according to the recommendations by [50]. All of the model assumptions were tested for conformity [51]. We calculated linear mixed models using ’lme4’ [52] package in ’R’. The estimated fixed effect coefficients were unstandardized.

Logistic mixed modelling to predict emotional arousal: The arousal ratings were divided into high arousal (>5) and low arousal (<=5). The high and low arousal categories were used as the response variable in the logistic mixed model. The interaction among ECG power in the midF range, EEG power, and familiarity was used as fixed effect. The dependency among measurements were controlled by considering random intercepts within participants. The regressions were fitted using GLMM with bobyqa as the optimizer, binomial distribution, and logit link. The coefficient calculation was performed using REML procedure. Significance and confidence interval were calculated using the Wald test. Homoscedasticity and independence requisites were met.

## 3. Results

### 3.1. Main Effect of ECG Power in Mid-Frequency Range

The ECG signal in the midF range showed a significant relationship with the EEG power. The regression results show region-specific laterality in brain activity. We observed a significant regression between ECG power with the EEG power in the right PO region in the alpha band (F(1, 220.69) = 4.91, β = −0.73, *p* = 0.028). The relationship is negative, which means one unit change in the ECG power related to a decrease in EEG power of the right PO region with the factor of 0.73.

### 3.2. The Influence of Familiarity on Cardiac–Brain Interaction during Emotion Processing

We calculated how the interaction between stimulus context familiarity and ECG power in the midF range relates to brain activity. Overall, we observed that familiarity influences the cardiac–brain interactions such that there was a stronger relationship between the ECG and EEG powers with an increase in familiarity.

**Figure 4:**
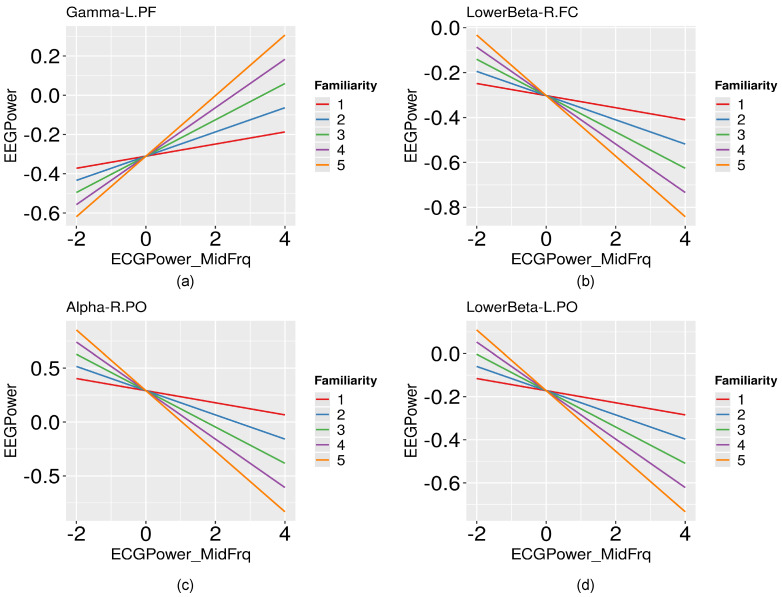
Cardiac-brain activity depends on stimulus context familiarity: Interaction plots depicting the dependence of the relationship between frequency specific standardized EEG power (y-axis) and standardized ECG power in midF range (x-axis) on familiarity. (**a**) Relationship between gamma activity in left PF and ECG power, (**b**) Relationship between lower beta activity in right FC and ECG power, (**c**) Relationship between alpha activity in right PO and ECG power, and (**d**) Relationship between lower beta activity in left PO and ECG power.

The interaction between familiarity and power of cardiac activity in the midF range was a significant predictor of the left PF activity in the gamma band (F(1, 162.41) = 3.58, β = 0.083, *p* = 0.043). The regression coefficient for EEG power as a function of ECG midF power was more positive for the more familiar compared to the less familiar emotional stimulus. When participants had an affective experience with a highly familiar stimulus, the gamma band activity in left PF increased with the increase in ECG power (Figure 4a).

The interaction between cardiac activity and familiarity had a significant effect on the lower beta activity in the right FC regions (F(1, 231.07) = 3.82, β = −0.079, *p* = 0.045). Once again, the cardiac and brain activity had a larger correlation with the high-familiar emotional videos in the right FC region. However, this correlation was negative; EEG power reduced with the increase in ECG power for highly familiar stimuli (Figure 4b).

The interaction between cardiac activity and familiarity was a significant predictor of lower beta activity in the left PO regions and alpha activity in the right PO regions (respectively, F(1, 231.42) = 4.77, β = −0.11, *p* = 0.03; F(1, 224.08) = 5.98, β = −0.22, *p* = 0.015). Again, a negative relationship was observed between cardiac activity and brain activity in the PO region for the increase in stimulus familiarity. For the highly familiar stimulus, the regression line is steeper (more negative) than for the less-familiar stimulus (Figure 4c,d).

### 3.3. Emotional Arousal Prediction

**Figure 5:**
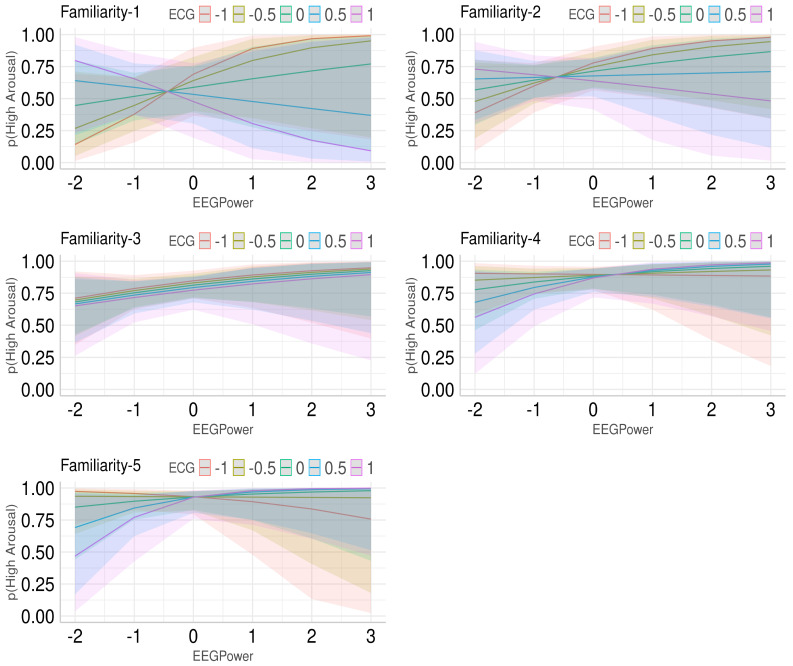
Interaction plots depicting the interaction among ECG power, EEG power in right PF regions, and familiarity in predicting arousal. Logistic mixed effect modelling was performed to predict arousal. X-axis shows standardized EEG power, y-axis shows the prediction probability for high arousal, and lines within the graphs depict ECG power (standardized). Each plot is for a different value of familiarity.

We observed the effect of interaction among ECG power in the midF range, gamma band power in the right PF region, and familiarity in predicting the odds of high arousal (odds = 1.427, β=0.557, SE = 0.181, z = 3.078, 95%CI = [0.202 0.912], *p* = 0.002). The interaction plots in Figure 5 show that for low familiarity, the probability for arousal being higher is greater when ECG power is lower and EEG power is high. Furthermore, when ECG power is high and EEG power is low, the prediction probability is greater for high arousal. On the other hand, when familiarity is higher, the predictions generally favour higher arousal. However, when ECG power is low, the probability for higher arousal is high with less EEG power but it does not decrease that much with the increase in EEG power. When the ECG power is high, the probability for higher arousal increases with EEG and at high values of EEG power, the ECG power does not matter. With medium levels of familiarity, the mean probability does not seem to depend too much on ECG power but it appears to increase variability.

**Figure 6:**
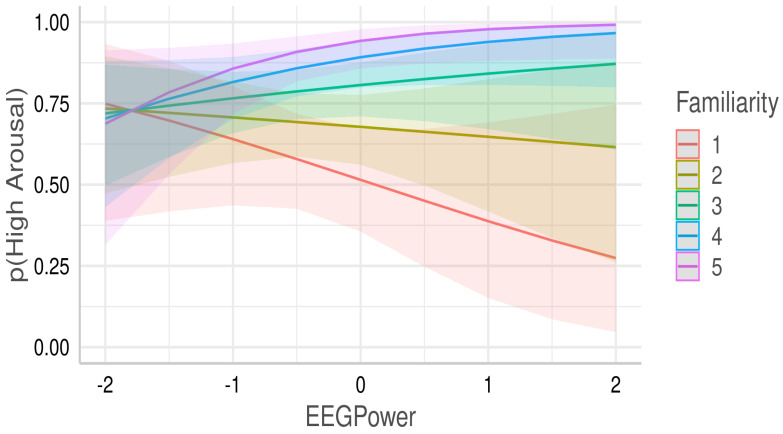
Interaction plot depicting the interaction between EEG power in right FC region and familiarity in predicting arousal. Logistic mixed effect modelling was performed to predict high arousal. X-axis shows standardized EEG power, y-axis shows the prediction probability for high arousal, and lines within the graphs depict prediction for different familiarity values.

In the right FC regions, the interaction between lower beta EEG power and familiarity predicts arousal (odds = 0.783, β=0.38, SE = 0.187, z = 2.033, 95%CI = [0.014 0.747], *p* = 0.042). The interaction between EEG power and familiarity is shown in Figure 6. When familiarity is low, an increase in EEG power is associated with a decrease in arousal and vice versa when familiarity is high.

The odds of predicting high arousal with alpha activity in the left FC regions are positive (odds = 0.974, β=0.407, SE = 0.19, z = 2.10, 95%CI = [0.027 0.786], *p* = 0.036). In addition, the lower beta activity in the left PO was also a significant predictor of high arousal (odds = 1.554, β=0.626, SE = 0.302, z = 2.072, 95%CI = [0.034 1.218], *p* = 0.038).

## 4. Discussion

Our primary aim in this exploratory study was to understand potential cardiac–brain interactions by probing the relationship between the ECG power in the midF range and oscillatory activity in different brain regions and how such interactions could be mediated by stimulus familiarity. We observed that the interaction between cardiac activity and familiarity was a significant predictor of gamma-band activity in the left PF region, lower beta activity in the right FC region, lower beta activity in the left PO region, and alpha activity in the right PO region. The relationship between EEG and ECG powers was stronger for stimuli with high context familiarity. Furthermore, we performed logistic mixed effect modelling to predict emotional arousal. We observed the prediction of emotional arousal on the frontal sites involving EEG power, ECG power, and familiarity. On the contrary, the posterior sites (left PO) had no significant interaction with familiarity in predicting emotional arousal.

The results show that ECG power in the midF range and average EEG power from the right FC regions in the lower beta band are inversely related as familiarity increases. Our results related to activity in the right FC regions are in line with a source localization study using emotional stimuli [53], which reported emotional modulation of HEP at FC electrodes (source localized to fronto-insulo-temporal networks) [53]. For positive and negative stimuli, activity in the right dorsal-anterior insula frontal operculum was observed [53]. The activity in the right frontocentral regions was also reported with static emotional faces [54]. Compared to neutral faces, a significantly reduced HEP activity was observed in the right FC sensors for the sad faces. Previous studies introduced context familiarity using cues to form an expectation about what picture is going to appear next [42,43,55]. Our results related to the effect of familiarity on the cardiac–brain interaction are in line with those cue-based studies. For instance, Refs. [42,43,55] reported more negative HEP amplitude in the right FC regions for the cued facial expressions (for angry as well as neutral) than the uncued facial expression. Thus, our results extend the validity of stronger, negative activity in the right FC regions with increasing context familiarity from static stimuli to naturalistic stimuli. The results with lower beta band power imply that attentional and pre-event memory formation processes associated with lower beta could be influenced by familiarity, resulting in changes in emotional arousal and interactions with cardiac activity [56,57,58].

When the effect of familiarity on cardiac activity was considered, we observed positively correlated gamma activity in the left PF regions. Synchronized gamma band activity in the PF regions was found for emotional information processing while watching emotional film clips, especially in the left frontal cortex [20]. Functional connectivity analysis in the gamma band also showed distinct functional networks for pleasant and unpleasant emotional experiences induced using pictures from the Chinese affective picture system. These functional networks involved the left prefrontal, parietal, and occipital regions [59]. Increased gamma power in the frontal regions as well as large-scale gamma phase synchronization across frontal regions for unpleasant stimuli was observed [23]. High gamma activity was also present during increased emotional responses in a cognitive reappraisal study with affective pictures [24].

Recently, the hierarchical modelling of interoceptive control modelled multiple parallel networks of insula with the prefrontal regions, which function in context-dependent learning and control of interoceptive responses, in order to represent emotions and visceral processes [60]. Although the model was simulated without taking laterality of the regions into account, the activity in the PF regions in our results is also a function of cardiac activity and familiarity. The correlated cardiac activity in the PF region is also in sync with the research on cardiac–brain interaction during the resting condition [16]. The activation/deactivation of the PF regions was shown to modulate the inter-beat-interval through the vagal control of the heart rate at rest [16]. It is possible that the activity in the PF regions in our study might be regulating cardiac activity through the vagal control of the heart rate during the emotional experience. Activity in the PF region was reported repeatedly in studies on the HEP during interoceptive or exteroceptive tasks [61,62] supporting the idea that activity in the PF region reflects the ongoing CNS processing of afferent/efferent cardiovascular information. The relationship was stronger for the stimulus with high familiarity than for the stimulus with less familiarity. Right PF gamma activity was argued to serve multiple roles including interoceptive control [63], reward expectation [64,65,66], cue-based stimulus categorization [65,67], and maintenance of relevant contents in working memory [68]. These functionalities seem essential for emotion related mechanisms and are consistent with familiarity dependent cardiac–brain interactions involving PF regions during the emotional experience.

We also observed that the power of the cardiac signal predicted the average EEG power in the right PO regions in the alpha band. In addition, the interaction between cardiac activity and familiarity significantly predicted lower beta activity in the left PO regions and alpha activity in the right PO regions. The influence of cardiac activity on PO regions is reported in experiments with naturalistic stimuli [2], the heartbeat counting task [22], and emotional stimuli [69]. For instance, PO alpha-band activity modulation was correlated with attention to the heart and the interoceptive heartbeat counting task [22]. With an ecologically valid experimental setting, the experience of arousal during the virtual reality experience of the rollercoaster ride was associated with the activity in the parietooccipital (PO) electrodes in the alpha band [2]. In a cue-based repetition suppression paradigm, in which a cue facilitated the context familiarity of emotional pictures, a significant reduction in alpha power over PO regions due to the expected facial expression was reported [55]. ERP amplitudes are greater over PO sites for repeated angry faces than for the neutral faces [43]. In sync with these studies, we also observed a stronger negative relationship with high familiarity stimuli than with low familiarity stimuli.

The rate of decrease in alpha power in the right PO regions is greater with the increasing power of the cardiac signal in the midF range for high familiarity than less-familiarity. This implies that, with high familiarity, cardiac activity is related to low alpha inhibition, i.e., more excitation in the right PO in comparison to less familiarity. In other words, the inhibitory activity in the right PO regions is a possible function of cardiac power and stimulus familiarity, such that with increasing familiarity and cardiac power, there is decreasing inhibitory activity in the right PO regions. On the other hand, the lower beta activity in the left PO and right FC regions also decreases with an increase in cardiac power. This rate of decrease is higher for high familiarity than for low familiarity.

The pre-event cortical activity in the alpha and lower beta band can be interpreted such that increased ongoing low beta activity reflects a memory forming state, which is likely to be moderated by modality-independent alpha specific attentional or inhibitory processes [57]. The inhibitory control activity was also reported to improve the resolution of uncertainty [70]. Hence, relatively high alpha power for less familiar stimuli might induce more inhibitory control to resolve uncertainty. In addition, the cardiac active inference model argues that cardiac activity modulates the activity in the sensory regions to seek more exteroceptive details in the case of uncertainty [15]. The memory formation functionality associated with the lower beta band is also modulated by the inhibitory activity and we postulate that the lower beta activity in the right FC and left PO might be related to pre-event memory formation, which is influenced by the cardiac activity and inhibitory control.

The odds of predicting high arousal with the interaction between lower beta activity in the right FC regions and the familiarity were significant. In addition, alpha activity in the left FC regions was also a significant predictor of emotional arousal. The lower beta activity in the left PO regions also had higher odds of predicting emotional arousal. Predominantly, alpha/lower beta activity in the left FC, right FC, and left posterior regions (PO) showed higher odds of predicting high arousal. The Alpha/lower beta activity with high arousal video clips [71] and highly arousing affective pictures from IAPS [56] was observed in parietal and occipital regions. These activities could be interpreted to reflect mirror neuron system activities, which were reported to be stronger for high arousal [71]. The mirror neuron system activity was also related to the interoceptive activity in the insula [72]. Arousing emotional stimuli modulated the synchronized activity in PO regions, with more desynchronized activity reported during high arousal [56]. Activity in the right FC regions is reported for interoceptive processing during the viewing of emotional faces. The source localization of the activity in the right FC was localized to the right anterior insula and right dorsal anterior cingulate cortex, which are involved in interoceptive processing [54] and the interoceptive activity in FC regions was associated with emotional arousal [73]. Hence, the dominant alpha/lower beta band activity in the frontal and posterior regions during the viewing of emotional film stimuli and the experience of emotions may reflect the essential role of inhibitory control, memory, interoceptive activity, and context in emotion processing.

Past observations suggest that activation of the right PF results in increased emotionality, an idea that is supported by the fact that when emotional stimuli were presented selectively to the right hemisphere, the raise in cortisol was greater [25]. An elevated cortisol level has been associated with heightened arousal [74]. However, our results indicate that arousal is dependent on a complex interaction among context familiarity, EEG power, and ECG power. The interaction is seen with gamma activity in the right PF region. The relationship between ECG and EEG powers in predicting arousal for low familiarity seems to be disjunctive and much less disjunctive with high familiarity. There seems to be less cardiac–brain interaction with stimuli of medium familiarity, with arousal being more dependent on EEG activity. The right prefrontal cortex is reported to be associated with the evaluation of the emotional experience and also with decision making. Damasio [75] reported that the decision functionality needed to take into account the emotional and somatic aspects (or bodily physiology aspect), which appears to be predominantly dependent upon the right PF [76].

The interaction with familiarity and ECG power in predicting arousal is not present in posterior (PO) regions, indicating that the context and interoceptive effects are predominant features of PF [77,78] during emotional arousal. A possible reason for such interactions could be due to activity in the insula [53,54]. The functionality of the insula is mostly attributed to the interoceptive sensation and multimodal integration [79] and not to the emotional evaluation. In addition, memory plays a role in emotional evaluation by injecting the context about the stimulus, which is compromised in dementia patients [80]. The gamma band activity in the right PF has been argued to be associated with the emotional memory [81,82]. Hence, we believe that the interaction between gamma activity in the right PF with familiarity and cardiac activity indicates an interaction among context, memory, cardiac activity, and emotional evaluation processes. The interesting dynamics among context familiarity, EEG power, and ECG power in predicting arousal need further study and theories of emotion need to consider this complex relationship to understand our emotional experience.

The results for EEG power as a function of familiarity and ECG power indicate a stronger relationship between EEG and ECG power across all significant interactions when familiarity is high. Context familiarity modulates cardiac–brain interoceptive interactions. Activity in some brain regions also predicts emotional arousal based on context familiarity. Our results are in line with the interoceptive theories, and constructive theories of emotions, which suggest that the context provides a descriptive framework to transform interoceptive bodily feelings into a situated emotional experience [27,35]. Our results are also consistent with appraisal theories of emotions, advocating familiarity as an essential dimension of emotional experience [29]. Previous studies have reported how context and interoceptive awareness can work as emotional priming to reduce the reaction time in recognizing facial expressions [39] and enhance the experience of emotional arousal [37,40]. Our results suggest that the coupling of the physiological changes with the emotional experience is mediated by context and it is possibly central to the interoceptive basis of emotional feelings [36].

In summary, this exploratory work attempts to understand the cardiac–brain dynamics and the role of context familiarity during the emotional experience as well as their potential influence on arousal using more naturalistic stimuli. The study shows potential relationships among cardiac activity, brain activity, self-reported familiarity, and arousal with multimedia films that are close to resembling the real-life dynamic experience of emotions. Second, the study indicates specific oscillations involved in cardiac–brain interactions prior to an emotional awareness mediated by familiarity. Third, instead of a cue manipulating context [83], our study incorporates familiarity with situations in emotional videos. Fourth, the results indicate that the possible influence of cardiac–brain interactions on arousal is dependent on context familiarity. Finally, the neurophysiological implications of our results suggest that band-specific activities in different brain regions in association with cardiac activity and familiarity of the emotional naturalistic stimuli could be related to the processes including attention, pre-event memory formation, interoception, and exteroception, which are essential constituents for the generation of emotional experience. We believe that the analysis and results presented in this work will lead to further studies seeking to understand the cardiac–brain dynamics underlying emotional experience.

## Data Availability

The data presented in this study are openly available in openneuro https://doi.org/10.18112/openneuro.ds003751.v1.0.3 (accessed on 13 April 2022).

## Abbreviations

The following abbreviations are used in this manuscript:

PF	Prefrontal
FC	Frontocentral
PO	Parietooccipital
midF	Mid-frequency
HEP	Heart Evoked Potential

## Appendix A

**Table A1:** Selected stimuli for EEG study from the stimuli dataset we created (for stimuli [45]; for related article [46]). The time duration of each stimulus is 60 s.

Stim Name	Target Emotion
ashayen	Adventorous
horror	Afraid
Anacondas_The_Hunt_for_the_Blood_Orchid_clip	Alarmed
Lage_Raho_Munnabhai_Only_the_Funny_Scenes_[360p]_2	Amused
anger_legend_of_baghat_singh	Angry
Divergent_Kiss_scene_clip	Aroused
Butterfly_Nets	Calm
Best_Horror_Kills_Ghost_Ship_Opening_scene	Disgust
Jai_Ho	Enthusiastic
SaddaHaq_1	Excited
MASOOM	Happy
cheerfulRang_1	Joyous
Crash_Saddest_scene	Melancholic
hate_lbs	Miserable
Madari_movie_of_best_scene_[360p]	Sad
Final_Race_of_Milkha_Singh_Career_[360p]	Triumphant
